# IRX5’s influence on macrophage polarization and outcome in papillary thyroid cancer

**DOI:** 10.3389/fonc.2024.1399484

**Published:** 2024-05-29

**Authors:** Lu Qin, Cheng Chen, Zhengwei Gui, Yun Jiang

**Affiliations:** ^1^ Department of Thyroid Vascular Surgery, Jingzhou Hospital Affiliated to Yangtze University, Jingzhou, ;China; ^2^ Department of Nuclear Medicine, Jingzhou Hospital Affiliated to Yangtze University, Jingzhou, ;China; ^3^ Tongji Hospital of Tongji Medical College of Huazhong University of Science and Technology, Department of Thyroid and Breast Surgery, Wuhan, ;China; ^4^ Department of Ultrasound, Hubei Hospital of Integrated Traditional Chinese and Western Medicines, Wuhan, ;China

**Keywords:** proliferation, tumor immune microenvironment, macrophage polarization, IRX5 gene, thyroid cancer

## Abstract

**Background:**

With a rise in recent years, thyroid cancer (TC) is the most prevalent hormonal cancer worldwide. It is essential to investigate the inherent variability at the molecular level and the immune environment within tumors of various thyroid cancer subtypes in order to identify potential targets for therapy and provide precise treatment for patients with thyroid adenocarcinoma.

**Methods:**

First, we analyzed the expression of IRX5 in pan-cancer and papillary thyroid carcinoma by bioinformatics methods and collected paired samples from our center for validation. Subsequently, we analyzed the significance of IRX5 on the prognosis and diagnosis of PTC. Next, we explored the possible mechanisms by which IRX5 affects the prognosis of thyroid cancer patients by GO/KEGG enrichment analysis, and further investigated the effect of IRX5 on immune infiltration of thyroid cancer. Ultimately, by conducting experiments on cells and animals, we were able to show how IRX5 impacts the aggressive characteristics of thyroid cancer cells and its influence on macrophages within the immune system of thyroid cancer.

**Results:**

In 11 malignant tumors, including PTC, IRX5 is overexpressed and associated with a poor prognosis. IRX5 may affect the prognosis of PTC through embryonic organ development, ossification, mesenchyme development, etc. Increased IRX5 expression decreases the presence of cytotoxic and Th17 cells in papillary thyroid cancer. IRX5 was highly expressed in different PTC cell lines, such as K-1 and TPC-1. Silencing IRX5 effectively halted the growth and movement of PTC cells while also decreasing M2 polarization and enhancing M1 polarization in tumor-associated macrophages.

**Conclusion:**

IRX5 could impact the outlook of individuals with PTC by stimulating the shift of macrophages to M2 in the immune surroundings of thyroid cancer growths, suggesting a potential new focus for treating thyroid cancer, particularly through immunotherapy.

## Introduction

Thyroid cancer is the most prevalent malignant tumor of the endocrine system globally, with a rising number of cases observed in the past few years (1–3). DTC, the most prevalent form of thyroid cancer, typically has a positive outlook. Nevertheless, managing patients with advanced DTC who experience recurrence, metastasis, and are resistant to iodine remains a significant clinical challenge. As the thyroid gland secretes thyroxine and other physiologically essential hormones, which are crucial for the growth and development of infants and children, there is a gradual trend toward a younger age of PTC onset. Thus, PTC threatens the health and development of an increasing number of people. It is essential to thoroughly investigate the inherent molecular diversity and immune environment of tumors in various types of thyroid cancer in order to identify potential therapeutic targets and improve the precision treatment and overall management of the disease.

IRX genes, similar to TALE-like homology box genes, are highly conserved among different species and play important roles in fundamental tissue development (4). IRX homology domain transcription factors were first identified in Drosophila, and play important roles in early as well as mid- and late stages of development. Previous research has demonstrated that IRX5 functions as a cancer-causing gene in liver cancer (5), tongue cancer (6), and lung cancer (7), as well as contributing to genetic instability in colorectal cancer (8). Yet, the involvement of IRX5 in papillary thyroid cancer remains undisclosed.

Immunotherapy is considered the most promising treatment for eliminating cancer, marking the beginning of a new era in cancer therapy (9). One of the ten traits of immune-evading tumors is the ability of tumor cells to instruct tumor-infiltrating leukocytes to shift from a pro-inflammatory to an anti-inflammatory state, preventing the elimination of tumor cells. Tumor-infiltrating macrophages (TIMs) originate from circulating monocyte progenitors and play a role in promoting tumor development, suppressing the immune system’s response to cancer, and facilitating the growth of new blood vessels (10, 11).High infiltration rates of TAMs are usually associated with a poor prognosis in solid tumors (12, 13). Macrophages have the ability to shift toward either M1/killer type macrophages, which are classically activated and have anti-tumor properties, or M2/restorative macrophages, which have anti-inflammatory effects but can also promote tumor growth (14). Promoting the polarization of TAMs toward M1 and reducing M2 polarization is one of the key research directions in tumor immunotherapy today (15–17).

## Materials and methods

### Clinical samples from patients

From June 2023 to December 2023, 10 paired cases of thyroid-like carcinoma (PTC) tissues and paracarcinoma tissues were collected at Tongji Hospital and stored in negative 80 degrees Celsius environment. Approval for the study was granted by the Ethics Committee at Tongji Hospital, with all participants providing written informed consent.

### Survival analysis in the TCGA database

PTC samples from the TCGA database were divided into two groups, high and low expression, according to IRX5 levels. The prognostic variances of these two were evaluated utilizing the Kaplan-Meier approach (http://www.kmplot.com) (18).

### Functional enrichment analysis

The R software’s clusterProfiler package was utilized to identify 735 genes with the strongest correlation to IRX5 co-expression in PTC (|FoldChange| > 1.5 and P < 0.05). The 708 genes associated with the prognosis of PTC were considered intersections. Subsequently, GO/KEGG pathway analysis were conducted on the resulting 66 genes, with visualization done using Graphpad Prism version 8.0.

### Cell culture and treatment

BCPAP, FTC133, K-1, and TPC-1 cell lines were acquired from the Wuhan Institute of Cell Biology in Wuhan, China.5% CO2 was added to a ThermoFisher incubator at 37°C.A dependable database is capable of identifying every acquired cell line through STR analysis.

### Pathological sample processing

Samples from tumors and adjacent tissues were preserved in 10% formalin, embedded in paraffin, cut into 5 mm slices, treated with dewaxing, rehydration, and microwaving. The IRX5 antibody from GeneTex (GTX52167) was used on the samples at 1°C for 30 minutes at ambient temperature, followed by staining the secondary antibodies with DAB substrate and then applying hematoxylin.

### RNA extraction and qRT-PCR

In order to isolate total RNA, we used TRIzol reagent along with IRX5 and GAPDH primers obtained from Tsingke Biotech. The primer sequences for IRX5 were as follows: forward GGGCTACAACTCGCACCTC and reverse CCCGTAAGGGTACGATCCCA.GAPDH forward - GGAGCGAGATCCCTCCAAAAT, reverse - GGCTGTTGTCATACTTCTCATGG.PCR was performed 40 times at 95°C for 5 minutes, then normalized to the internal control and analyzed using a 2-ΔΔCT approach.

### CCK8 assay

Optical density in each group was measured using an enzyme marker at 450 nm. Each group digested the cells and resuspended them in full culture media. Cell growth was measured using the CCK-8 assay at 24, 48, 72, and 96 hours.

### Colony-formation assay

Following inoculation of 5,000 cells into six-well plates, colonies were dyed with crystal violet after being fixed for 10 minutes in 4% polyacetal. Photographs and tallies of colonies were captured.

### Transwell assay

A transwell upper chamber (Corning, USA) contains 20,000 cells that were wiped from its top surface after 24 hours at 37°C. Crystal violet stain was applied for 10 minutes to the chamber bottom, and migrating cells were counted and imaged after that.

### Scratch test

Healthy K-1 and TPC-1 thyroid cancer cells were used to incubate IBIDI two-well culture inserts for 36 hours in 24-well plates. The inserts were delicately extracted from the untouched surface using forceps, then each well was filled with 1 mL of media with low serum content. Cell movement was detected using a light microscope on days 0 and 1 following removal of the inserts.

### Tumor xenograft

12 female nude mice without thymus were divided into two groups, each consisting of 8-week-old mice with an average weight of 24 g.A total of 1 × 106 K1 cells were subcutaneously implanted into the lateral abdomen of the mice. Every other day, the xenografts’ dimensions were assessed. The ultimate size of the xenografts was determined by calculating V = 0.5 × L (tumor length) × W2 (tumor width). After a period of 20 days, every nude mouse was given anesthesia until they died, and then the tumors that had been transplanted were removed and weighed. The Animal Ethics Committee of Jingzhou Central Hospital approved and oversaw our study.

### Immune cell infiltration

Analysis of immune cell infiltration in PTC was conducted by using the GSVA package in R. The results were derived from the ssGSEA package. After categorizing 24 immune cells and consulting past research, the level of IRX5 expression in TCGA-THCA samples was measured according to the extent of immune cell infiltration (high or low).

### Immunofluorescence microscopy

Actin and nuclei were observed by exposing cells to paraformaldehyde solution for 15 minutes and staining with rhodamine ghost pen cyclic peptide at a concentration of 2.5 units/ml and DAPI. Fluorescence microscopy was then used to examine the stained cells.

### ELISA

An ELISA test was conducted to measure the levels of specific cytokines in the culture medium obtained from the lower chamber of a coculture of PTC cells and macrophages. Using an enzyme marker, we determined the absorbance at 450 nm by calculating a standard curve and expressing the result in picograms per milliliter.

## Results

### IRX5 expression analysis

**Figure 1:**
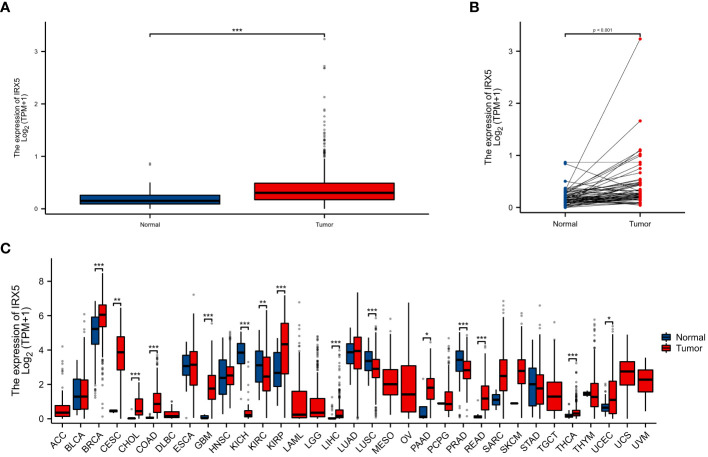
The expression difference of IRX5 in cancer tissue and normal tissue. **(A)** Expression of IRX5 in unpaired thyroid cancer samples in TCGA-THTC database. **(B)** Expression of IRX5 in paired thyroid cancer samples in TCGA-THTC database. **(C)** Expression of IRX5 in pan-cancer and adjacent normal tissues in TCGA and GTEx databases. Data were shown as mean ± SD. *p < 0.05, **p < 0.01, ***p < 0.001.

Unpaired (**Figure 1A**) and paired samples (**Figure 1B**) of the TCGA thyroid cancer cohort showed significantly high expression.

As a result, we investigated IRX5 expression in both the TCGA and GTEx pancancer databases and found that it was higher in 11 tumors compared to normal tissues (**Figure 1C**).

**Figure 2:**
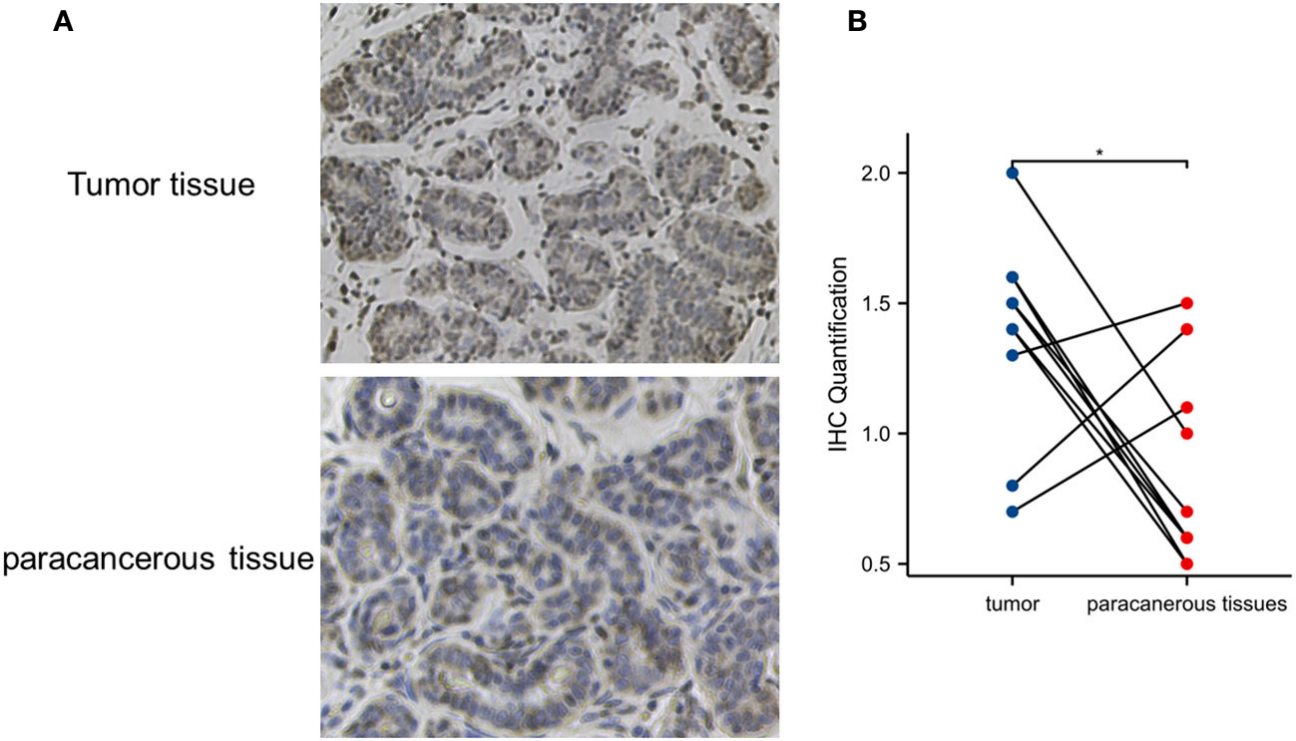
Verification of IRX5 overexpression in thyroid cancer in our center specimen. **(A)** Typical cancer and paracancer IRX5 immunohistochemical images. **(B)** Quantitative IRX5 immunohistochemical analysis of 10 paired specimens. *p < 0.05.

To verify the overexpression of IRX5 in PTC, we collected 10 pairs of paired PTC samples from our center, typical immunohistochemistry results are shown in **Figure 2A**, and quantitative analysis of the paired samples are shown in **Figure 2B**, which showed that IRX5 was overexpressed in the PTC cancer foci, which was in line with the results of bioinformatics analysis.

### The correlation between the expression of IRX5 and the outlook for individuals with PTC

**Figure 3:**
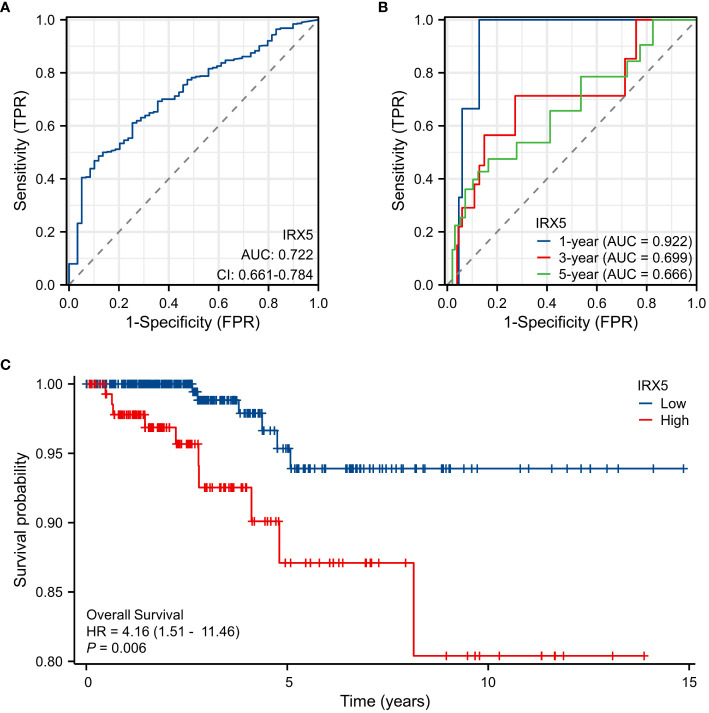
Expression of IRX5 and prognosis of thyroid cancer patients. **(A)** Diagnostic ROC curve of IRX5. **(B)** Time-dependent ROC curve of IRX5. **(C)** OS of thyroid cancer patients based on IRX5 expression level.

Diagnostic ROC curve of IRX5 in PTC (**Figure 3A**) AUC=0.722 (CI=0.661–0.784) and time-dependent ROC curve (**Figure 3B**) AUC=0.922 (1year), 0.699 (3year), 0.666 (5year). The above results suggest that IRX5 has good predictive value for PTC. In the TCGA cohort, we examined the association between IRX5 expression and OS (**Figure 3C**) to test whether it can be used to predict cancer patient prognosis. High levels of IRX5 were linked to unfavorable outcomes in PTC, with a hazard ratio of 4.16 and a significance level of 0.006.

### Correlation and enrichment analyses

**Figure 4:**
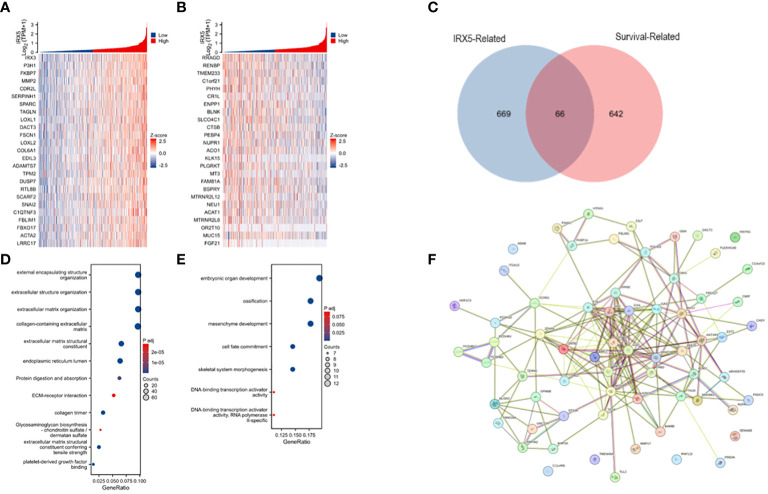
Analysis of IRX5-related enrichment pathways. **(A)** Heatmap of TOP25 genes positively associated with IRX5 co-expression. **(B)** Heatmap of TOP25 genes negatively associated with IRX5 co-expression. **(C)** Wayne plot of IRX5 co-expressed genes taking intersection with PTC survival related genes. **(D)** GO/KEGG analysis of IRX5 co-expressed genes. **(E)** GO/KEGG analysis of intersecting genes. **(F)** Protein interactions network of intersecting genes.

Analysis of TCGA-THCA data was conducted with the statistical package in R software, identifying the top 50 molecules showing the strongest positive or negative correlation with IRX5 co-expression for heatmap visualization (**Figures 4A, B**). The top genes correlated with IRX5 co-expression in PTC (735 genes) were identified using specific criteria and compared with PTC prognosis-related genes (708 genes). A total of 66 genes were selected for further analysis, revealing potential pathways linking IRX5 to PTC prognosis, including embryonic organ development, ossification, and mesenchyme development (**Figures 4C–E**). The protein interaction network of these 66 molecules is shown in **Figure 4F**.

### Expression of IRX5 and immune cell infiltration

**Figure 5:**
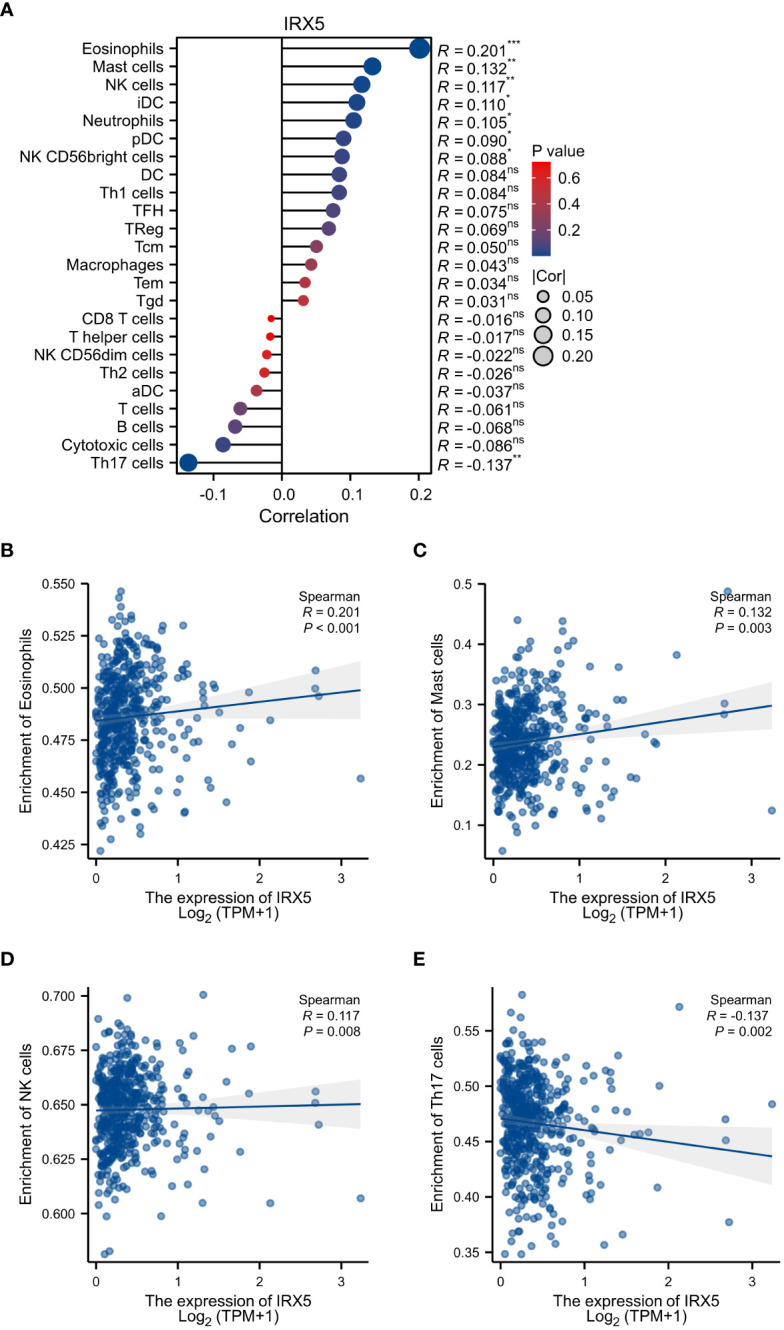
Associated between IRX5 with immune cell infiltration. **(A)** Correlation between the expression level of IRX5 and various immune cell infiltration. **(B)** Correlation between IRX5 expression and Eosinophils. **(C)** Correlation between IRX5 expression and Mast cells. **(D)** Correlation between IRX5 expression and NK cells. **(E)** correlation between IRX5 expression and Th17 cells. ns, not statistically.

Then, we examined the immune cell infiltration of PTC patients (**Figure 5A**). In IRX5 overexpressing PTC, the infiltration of Eosinophils (**Figure 5B**), Mast cells (**Figure 5C**) and NK cells (**Figure 5D**) was elevated, while the infiltration of Th17 cells (**Figure 5E**) and Cytotoxic cells was decreased.

### IRX5 knockdown inhibited PTC cells’ malignant behavior

**Figure 6:**
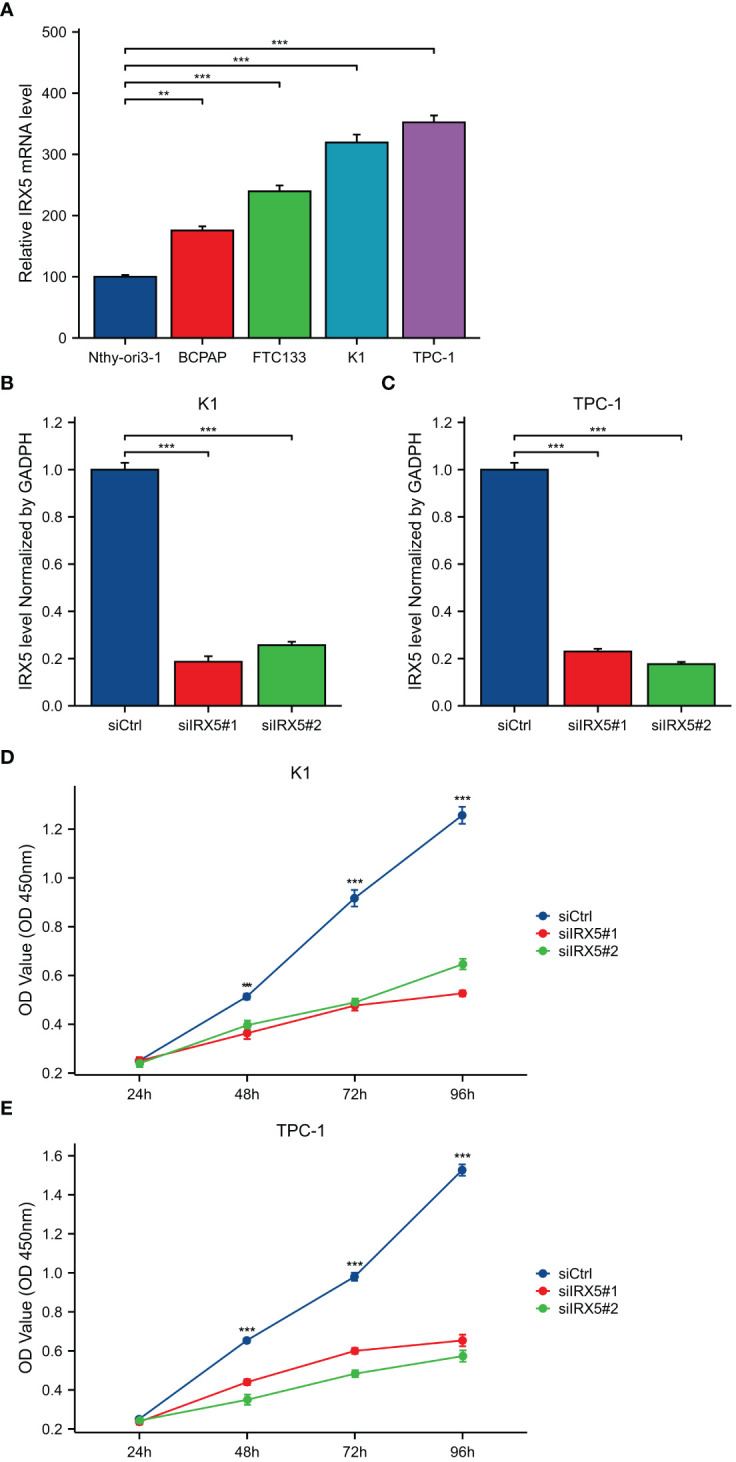
Expression and knockdown of IRX5 in various cell lines and CCK8 cell proliferation experiment. **(A)** IRX5 expression in Nthy-ori3–1, BCPAP, FTC-133, K-1 and TPC-1 cell lines. **(B)** IRX5 knockdown efficiency of two siRNA in K-1 cell lines. **(C)** IRX5 knockdown of two siRNA in TPC-1 cell lines Efficiency. **(D, E)** Cell proliferation in two siRNA knockout groups and control groups in K-1 and TPC-1 cell lines. **p < 0.01, ***p < 0.001.

**Figure 7:**
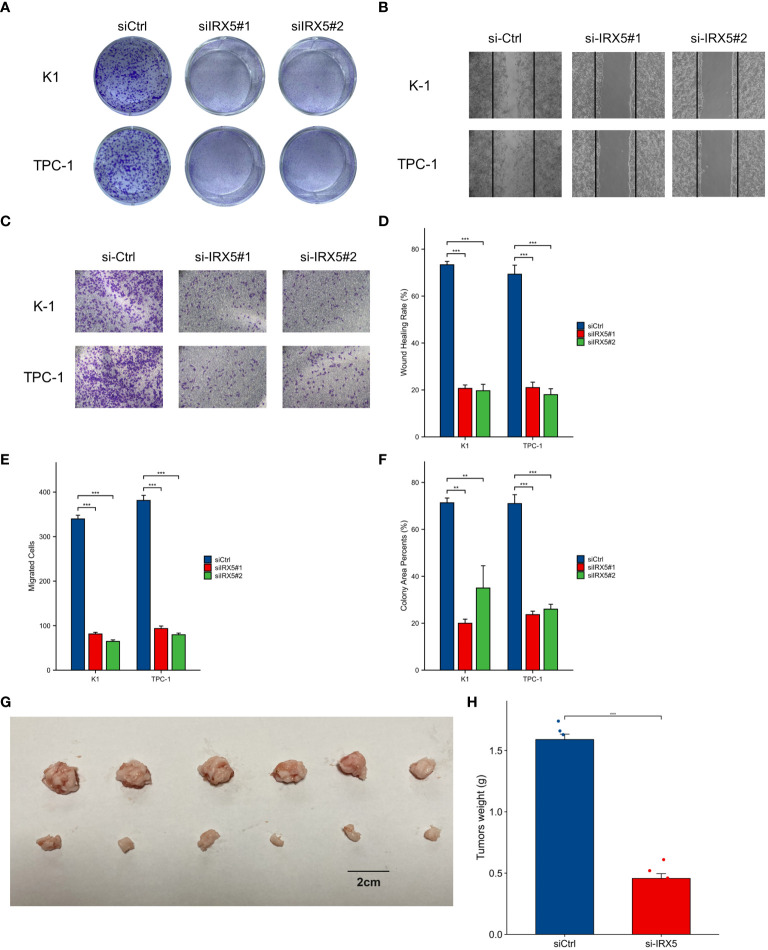
Cellular and animal experiments to validate the effect of IRX5 on PTC cells. **(A)** Clone formation of control group and two siRNA knockout groups in K-1 and TPC-1 cell lines. **(B)** Scratch test images of control group and two siRNA knockout groups in K-1 and TPC-1 cell lines. **(C)** Transwell images of control group and two siRNA knockout groups in K-1 and TPC-1 cell lines. **(D)** Quantitative analysis of clone formation experiment. **(E)** Quantitative analysis of scratch experiment. All assays were independently repeated at least three times. **(F)** Quantitative analysis of transwell experiment. **(G)** Growth of transplanted tumors in nude mice injected with K-1 cells and si-IRX5 K-1 cells. **(H)** Weight of grafted tumors. Data are presented as the mean ± SD. **p < 0.01, ***p < 0.001.

BCPAP, FTC133, K-1, and TPC-1 cells exhibited high levels of IRX5 expression, unlike Nthy-ori3–1 cells, which are a model of healthy thyroid cells. (**Figure 6A**). To investigate the impact of IRX5 on the growth of PTC cells, we employed two siRNAs targeting IRX5 to suppress its expression in K-1 and TPC-1 cells. (**Figures 6B, C**) After measuring cell proliferation with CCK8 assays, the results showed that IRX5 knockdown significantly reduced proliferation rates in both cell lines (**Figures 6D, E**). The negative effect of IRX5 on PTC cell growth was confirmed through a colony formation test, as shown in **Figure 7A**. The outcomes of the transwell experiment and scratch test also indicated a significant decrease in the migratory capacity of PTC cells following the suppression of IRX5 (**Figures 7B, C**). We conducted quantitative analyses on scratch, transwell, and clone generation assays (**Figures 7D–F**). Growth of transplanted tumors in nude mice injected with K-1 cells and si-IRX5 K-1 cells and growth curve of grafted tumor volume are showed in **Figures 7G, H**. To sum up, IRX5 triggers biological activities that result in cancer, including movement, growth, and penetration.

### Effect of IRX5 knockdown on tumor-associated macrophages in PTC

**Figure 8:**
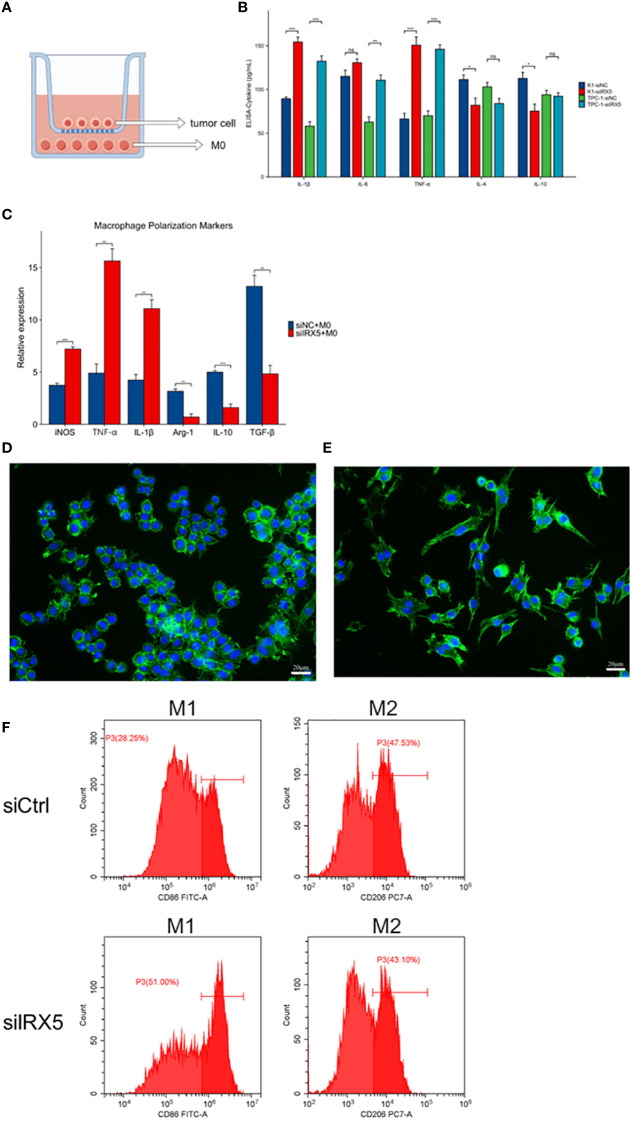
Effect of IRX5 knockdown on thyroid cancer tumor-associated macrophages. **(A)** Schematic diagram of the co-culture model of thyroid cancer cells and macrophages. **(B)** Cytokine content (ELISA) of culture fluid in co-culture chambers. **(C)** Macrophage polarization marker expression. **(D)** Immunofluorescence graph of macrophage morphology (si-IRX5). **(E)** Immunofluorescence graph of macrophage morphology (si-Ctrl). **(F)** Detection of macrophage polarization in mouse tumors by flow cytometry. * p < 0.05, **p < 0.01, ***p < 0.001. ns, not statistically.

To model the tumor immune environment where tumor cells interact with macrophages associated with tumors. We constructed a PTC co-culture model with macrophages as shown in **Figure 8A**. Transwell chambers with a pore diameter of 1 μm were chosen, with K-1 and TPC-1 cells placed in the top chamber and RAW264.7 cells in M0 condition placed in the bottom chamber. After 36 hours of co-culture, the cell cultures in the lower chamber were collected for cytokine detection by ELISA assay. **Figure 8B** displays that macrophages co-cultured with PTC from the IRX5 knockdown group released higher levels of IL-1β, IL-6, and TNF-α, while producing lower amounts of IL-4 and IL-10 compared to the control group. RNA was extracted from lower chamber macrophages and analyzed for macrophage polarization markers. Results indicated increased levels of iNOS, TNF-α, and IL-1β in macrophages co-cultured with PTC from the IRX5 knockdown group compared to the control group, while levels of Arg-1, IL-10, and TGF-β were reduced (**Figure 8C**). Immunofluorescence photographs showed that macrophages co-cultured with the IRX5 (**Figure 8D**) group and NC (**Figure 8E**) knockdown group were polarized like M2 and M1 direction, respectively (**Figure 8F**).

**Figure 9:**
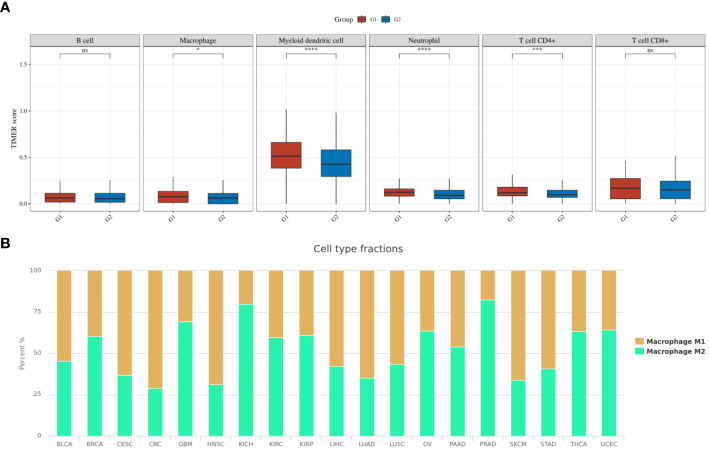
Immune cell infiltration in TCGA-THCA. **(A)** TIMER scores related to IRX5 expression in TCGA-THCA (the cohort was divided into two groups by the 50% median IRX5 expression. G1:IRX5 high expression group, G2:IRX5 low expression group) **(B)** TCIA database analysis of the M1 to M2 ratio of TAM in various malignancies. ns, not statistically. *p < 0.05, ***p < 0.001, ****p < 0.0001.

To further explore the impact of macrophage infiltration and polarization on the prognosis of PTC patients, we used the TIMER algorithm of the immunedeconv software package to analyze the immune infiltration of PTC patients with different IRX5 expression in the TCGA database, as shown in **Figure 9A**. Macrophages, Myeloid dendritic cells, Neutrophil and CD4+ T cells were elevated in our IRX5 high expressing PTC. We then analyzed the TCGA data using the cell type scores from the Cancer Immunology Database (TCIA), as shown in **Figure 9B**, and in THCA, the macrophage M2 percentage was the 6th highest (63%) among the various malignancies.

## Discussion

Thyroid cancer rates have been increasing worldwide for the last four decades. Based on the 2020 WHO GLOBOCAN cancer incidence and mortality database, thyroid cancer ranks ninth worldwide in terms of cancer incidence (19). Molecular subtypes of PTC have been identified, including BRAF-like and RAS-like, which are both associated with genetic mutations and tumorigenesis (20, 21). Several gene mutations at the molecular level have been linked to PTC malignancy and clinicopathological characteristics, however, none of these mutated genes have been proven to assist in treatment or confirm the diagnosis at this time. Therefore, the discovery of new causative genes will provide new hope for precision treatment of thyroid cancer.

IRX5, part of the IRX gene family, was discovered to be upregulated in several types of cancers, such as thyroid cancer. Samples were gathered from our facility for further confirmation. Meanwhile, IRX5 overexpressed PTC patients have a worse prognosis. The intersection of IRX5 co-expressed genes and PTC survival-related genes was taken and analyzed by GO/KEGG enrichment, which led to the possible pathways by which IRX5 affects the prognosis of PTC. The results of cellular and animal experiments further demonstrated that IRX5 overexpression promotes malignant biological behaviors such as proliferation and migration of PTC cells.

In solid tumors, there are various types of cells, including malignant cells, cancer stem cells, fibroblasts, stromal cells, and immune cells from both innate and adaptive immunity (22). For tumor growth to occur, it is critical for cancer cells and immune cells to communicate within the tumor environment. A macrophage is a versatile cell with several functions that are crucial for both innate and adaptive immunity in vertebrates. Tumor-infiltrating monocytes from the peripheral blood are responsible for producing TAM. Current studies have shown that TAMs are constantly switching between M1 and M2 types (23, 24). M1-macrophages exhibit antitumor properties by identifying and eliminating tumor cells through two distinct methods: direct cytotoxicity and antibody-dependent cell-mediated cytotoxicity. On the other hand, M2-macrophages are categorized into various subtypes with different functions: M2a aids in tissue fibrosis, M2b supports tumor advancement, M2c contributes to tissue reconstruction, and M2d stimulates angiogenesis (25–27).

Tumor-associated macrophages (TAMs) are immune cells that have the ability to infiltrate thyroid tumors and make up to half of the tumor’s total volume. In contrast, in thyroid cancer, TAMs predominantly exhibit an M2 phenotype. Thyroid cancer cells and M2-macrophages have the ability to stimulate one another. M2-TAMs aid in the dedifferentiation, growth, and spread of TC by releasing Wnt1 and Wnt3 ligands, which trigger the Wnt signaling pathway and enhance β-catenin activation. Thyroid cancer cells have the ability to cause macrophage polarization, which then influences tumor cells, resulting in the advancement and spread of the tumor (28). Furthermore, TAM polarization is influenced by genetic mutations like the BRAFV600E mutation, resulting in a higher proportion of M2-TAMs and enhancing tumor development (29).

Our current research revealed heightened eosinophil and NK cell penetration and reduced Th17 cells in PTC with IRX5 overexpression. Research has shown that eosinophils have the ability to display various receptors on their exterior that can communicate with NK cells, such as ntb-a, 2B4, CD84, CD58, IRp60, CD48, and LFA-1 (ICAM-3) (30, 31). During prolonged periods of inflammation, eosinophils suppress the cytotoxic function of natural killer cells, leading to an elevated tumor burden (32). Th17 cells are characterized by being CD4-positive T lymphocytes that produce high levels of interleukin 17A (IL-17A) and contain the transcription factor retinoic acid receptor-associated orphan receptor γT (33, 34). These cells can develop into either immunosuppressive Treg cells (35, 36) or pro-inflammatory Th1 cells (37) under certain circumstances, both of which exhibit notable anti-tumor effects (38, 39). This mirrors the polarization seen in tumor-associated macrophages. The involvement of Th17 lymphocytes in cancer is still a topic of much debate.

By constructing a co-culture model of thyroid tumor cells and macrophages, we found that IRX5 knockdown thyroid cancer cells could induce macrophages to secrete multiple pro-inflammatory cytokines and polarize toward anti-tumor M1, which may be a new direction for thyroid cancer immunotherapy. Interestingly, in the course of macrophage M1 polarization, the pro-inflammatory cytokines within the tumor’s immune microenvironment have the ability to prompt the transformation of Th17 cells into Th1 cells, potentially enhancing the efficacy of immunotherapy. This is the focus of our next research.

Among the 19 malignant tumors included in the TCGA database, the macrophage M2/M1 ratio in THCA ranked 6th (63%), and therefore, regulating macrophage polarization toward M1 has significant potential in THCA treatment. Previously, it was reported that P53 is an important regulator of macrophage polarization (40, 41), and in hepatocellular carcinoma, IRX5 may promote hepatocellular carcinoma proliferation and inhibit its apoptosis by regulating the TP53 signaling pathway (42). Therefore, we hypothesized that in PTC, knockdown of IRX5 may inhibit the development of PTC by suppressing the TP53 signaling pathway, thereby contributing to the polarization of TAM toward M1, which ultimately inhibits the development of PTC.

Although we identified the potential PTC oncogene IRX5 and verified its relationship with tumor-associated macrophage polarization, the *in vivo* environment is far more complex compared to *in vitro* models. A large number of individuals with thyroid cancer have connections to chronic lymphocytic thyroiditis or Graves’ disease, conditions that change the presence of immune cells in the thyroid. It remains uncertain if the intricate immune cell groupings will impact the effectiveness of immunotherapy for these patients.

## Data availability statement

The original contributions presented in the study are included in the article/supplementary material, further inquiries can be directed to the corresponding author/s.

## Ethics statement

The studies involving humans were approved by Ethics Committee of Tongji hospital (TJ20230311) The studies were conducted in accordance with the local legislation and institutional requirements. The participants provided their written informed consent to participate in this study. The animal study was approved by Ethics Committee of Tongji hospital. (TJ20230412) The study was conducted in accordance with the local legislation and institutional requirements.

## Author contributions

LQ: Investigation, Methodology, Project administration, Resources, Software, Visualization, Writing – original draft, Writing – review & editing. CC: Conceptualization, Data curation, Investigation, Project administration, Writing – original draft. ZG: Writing – review & editing, Validation. YJ: Formal analysis, Resources, Software, Supervision, Validation, Visualization, Writing – original draft, Writing – review & editing.
